# Molecular characterization and antimicrobial susceptibility of *Acinetobacter baumannii* isolates obtained from two hospital outbreaks in Los Angeles County, California, USA

**DOI:** 10.1186/s12879-016-1526-y

**Published:** 2016-05-04

**Authors:** Wayne A. Warner, Shan N. Kuang, Rina Hernandez, Melissa C. Chong, Peter J. Ewing, Jen Fleischer, Jia Meng, Sheena Chu, Dawn Terashita, L’Tanya English, Wangxue Chen, H. Howard Xu

**Affiliations:** Department of Biological Sciences, California State University, Los Angeles, Los Angeles, California 90032 USA; Los Angeles County Department of Public Health, Public Health Laboratory, Los Angeles, CA 90242 USA; Los Angeles County Department of Public Health, Los Angeles, CA 90012 USA; Human Health Therapeutics, National Research Council Canada, Ottawa, ON K1A 0R6 Canada

**Keywords:** *Acinetobacter baumannii*, Nosocomial outbreak, Epidemiology, Antimicrobial susceptibility, Mechanism of resistance

## Abstract

**Background:**

Antibiotic resistant strains of *Acinetobacter baumannii* have been responsible for an increasing number of nosocomial infections including bacteremia and ventilator-associated pneumonia. In this study, we analyzed 38 isolates of *A. baumannii* obtained from two hospital outbreaks in Los Angeles County for the molecular epidemiology, antimicrobial susceptibility and resistance determinants.

**Methods:**

Pulsed field gel electrophoresis, tri-locus multiplex PCR and multi-locus sequence typing (Pasteur scheme) were used to examine clonal relationships of the outbreak isolates. Broth microdilution method was used to determine antimicrobial susceptibility of these isolates. PCR and subsequent DNA sequencing were employed to characterize antibiotic resistance genetic determinants.

**Results:**

Trilocus multiplex PCR showed these isolates belong to Global Clones I and II, which were confirmed to ST1 and ST2, respectively, by multi-locus sequence typing. Pulsed field gel electrophoresis analysis identified two clonal clusters, one with 20 isolates (Global Clone I) and the other with nine (Global Clone II), which dominated the two outbreaks. Antimicrobial susceptibility testing using 14 antibiotics indicated that all isolates were resistant to antibiotics belonging to four or more categories of antimicrobial agents. In particular, over three fourth of 38 isolates were found to be resistant to both imipenem and meropenem. Additionally, all isolates were found to be resistant to piperacillin, four cephalosporin antibiotics, ciprofloxacin and levofloxacin. Resistance phenotypes of these strains to fluoroquinolones were correlated with point mutations in *gyrA* and *parC* genes that render reduced affinity to target proteins. IS*Aba1* was detected immediately upstream of the *bla*_OXA–23_ gene present in those isolates that were found to be resistant to both carbapenems. Class 1 integron-associated resistance gene cassettes appear to contribute to resistance to aminoglycoside antibiotics.

**Conclusion:**

The two outbreaks were found to be dominated by two clonal clusters of *A. baumannii* belonging to MLST ST1 and ST2. All isolates were resistant to antibiotics of at least four categories of antimicrobial agents, and their antimicrobial susceptibility profiles correlate well with genetic determinants. The results of this study will facilitate our understanding of the molecular epidemiology, antimicrobial susceptibility and mechanisms of resistance of *A. baumannii* obtained from Los Angeles hospitals.

## Background

*Acinetobacter baumannii*, an emerging opportunistic pathogen, is responsible for a significant proportion of nosocomial infections including urinary tract infections, endocarditis, surgical site infections, meningitis, septicemia, and ventilator associated pneumonia among intensive care unit patients in hospitals [1–5]. Additionally, *A. baumannii* is recognized as an increasingly important cause of community-acquired pneumonia and other infections [6–11]. Currently, many nosocomial clinical isolates of *A. baumannii* exhibit antimicrobial resistance towards most or all major classes of antibiotics including β-lactams, aminoglycosides, fluoroquinolones (FQs), chloramphenicols, tetracyclines and rifampin [12–16]. Four decades ago, these antibiotics were effective in treating infections caused by this bacterium. In particular, multidrug resistant clinical isolates of this bacterium have been reported as infectious agents in many soldiers wounded in Afghanistan and Iraq [17–22].

A few years ago, we examined the phenotypic and molecular characteristics of 20 representative outbreak isolates of *A. baumannii* obtained from Los Angeles County (LAC) hospitals [14]. Subsequently, in vivo virulence screens identified among these isolates a hypervirulent strain (LAC-4) which was used to develop a mouse model of *A. baumannii*-associated pneumonia [23]. Recently, we reported the complete genome of LAC-4 [24]. In this study, we determined antimicrobial susceptibility and performed detailed molecular analyses of 38 additional isolates of *A. baumannii* derived from two hospital outbreaks in Los Angeles County. Our report provides critical insight into the development of antimicrobial drug resistance, clonal dissemination and evolution of clinical isolates of *A. baumannii* in LA County hospitals.

## Methods

### Epidemiology and definitions of case and control patients/isolates

*A. baumannii* isolates were collected as part of two separate healthcare-associated outbreak investigations that occurred in two Los Angeles County hospitals in 2007 and 2009 respectively. Hospital A (Outbreak X) is a large tertiary care teaching facility that provides general and specialized medical services. The outbreak occurred in the burn unit, a six-bed unit with two rooms located across the hall from each other. Hospital B (Outbreak Y) is a medium sized tertiary care facility that provides general and specialized medical services. The outbreak occurred in the medical intensive care unit. In both outbreaks isolates from case patients (thus the isolates are called case isolates) were cultured after the onset of new symptoms, e.g. fever, increased sputum. Control patients had *A. baumannii* cultures during the outbreak period from the same facility and, based on epidemiologic data, these cultures were determined to be unrelated to the outbreak (thus these isolates are called control isolates). Control isolates were used as a baseline for comparison of current strains circulating within the facility. Environmental cultures were collected from multiple unit surfaces of Hospital B, including patient room sinks, faucets and door handles as well as ventilator supplies and equipment. Environmental Cultures were not collected from Hospital A. *A. baumannii* isolates are shown in Table 1.

**Table 1:** *A. baumannii* isolates, nature of samples (case vs control), their collection dates, sites, source of outbreaks and PFGE type

Isolate No.	Case/Control	Collection Date	Specimen Source	Outbreak Code	No. of Band Differences	PFGE Type
LAC-201	Case 1	12/19/2007	BAL	X	0	X–A
LAC–202	Case 1	12/14/2007	Blood	X	0	X–A
LAC–203	Case 2	12/29/2007	Tip	X	0	X–A
LAC–204	Case 2	12/30/2007	ETT	X	0	X–A
LAC–205	Case 3	12/8/2007	Tip	X	0	X–A
LAC–206	Case 3	11/29/2007	Tip	X	0	X–A
LAC–207	Case 3	12/8/2007	BAL	X	0	X–A
LAC–208	Case 4	11/20/2007	BAL	X	0	X–A
LAC–209	Case 4	12/1/2007	Blood	X	1	X–A1
LAC–210	Control 1	12/23/2007	Wound	X	3	X–A2
LAC–211	Control 1	12/29/2007	Blood	X	3	X–A2
LAC–212	Case 5	12/22/2007	BAL	X	3	X–A3
LAC–213	Case 5	12/30/2007	BAL	X	3	X–A3
LAC–214	Case 5	12/21/2007	ETT	X	3	X–A3
LAC–215	Case 6	12/8/2007	Urine	X	3	X–A3
LAC–216	Case 6	12/20/2007	Blood	X	3	X–A3
LAC–217	Control 2	12/4/2007	Blood	X	4	X–A4
LAC–218	Control 2	11/29/2007	Resp	X	4	X–A4
LAC–219	Control 3	1/4/2008	Resp	X	3	X–A5
LAC–220	Control 3	1/3/2008	Blood	X	4	X–A6
LAC–221	Control 4	10/19/2007	BAL	X	>7	X–B
LAC–222	Control 4	11/10/2007	Blood	X	>7	X–B
LAC–223	Control 5	10/29/2007	BAL	X	>7	X–B1
LAC–224	Control 6	11/16/2007	Tip	X	>7	X–C
LAC–225	Control 6	11/22/2007	Resp	X	>7	X–C
LAC–226	Control 5	10/24/2007	Wound	X	>7	X–D
LAC–227	Case 1	8/4/2009	Sputum	Y	0	Y–A
LAC–228	Case 2	8/24/2009	Sputum, Expectorated	Y	0	Y–A
LAC–229	Case 3	8/31/2009	Sputum	Y	0	Y–A
LAC–230	Case 4	9/3/2009	Sputum	Y	0	Y–A
LAC–231	Environmental	9/15/2009	Environmental	Y	0	Y–A
LAC–232	Environmental	9/15/2009	Environmental	Y	0	Y–A
LAC–233	Environmental	9/15/2009	Environmental	Y	0	Y–A
LAC–234	Case 5	8/6/2009	Sputum	Y	1	Y–A1
LAC–235	Control 1	9/6/2009	Wound	Y	1	Y–A2
LAC–236	Case 6	8/4/2009	Bronchoscopy	Y	>7	Y–B
LAC–237	Case 7	8/6/2009	Sputum	Y	>7	Y–B
LAC–238	Case 8	8/3/2009	Wound	Y	>7	Y–C

*Abbreviations*: *BAL* bronchoalveolar lavage, *ETT* endotracheal tube, *resp* respiratory, *tip* catheter tip

### Additional bacterial strains

Quality control strains used in antimicrobial susceptibility testing [*Escherichia coli* (ATCC #25922) and *Pseudomonas aeruginosa* (ATCC #27853)] were purchased from American Type Culture Collection (ATCC, Manassas, VA). Additionally, *A. baumannii* strains AYE [12] and CCF-9 [25] were used as positive controls for Global Clones I and II (formerly known as International Clones I and II), respectively. All strains were stored in 20 % glycerol at – 80 °C.

### Species-level confirmation of *A. baumannii* isolates

All isolates were previously determined to be *A. baumannii* by hospital clinical laboratories. Species of these isolates were further confirmed based on sequence analysis of 16S–23S rRNA gene intergenic spacer (ITS) regions using modified methods [25] based on Chang and coworkers [26].

### Pulsed field gel electrophoresis (PFGE)

Bacterial genomic DNA plugs were made from *A. baumannii* isolates according to PulseNet USA standardized laboratory protocol (http://www.cdc.gov/pulsenet/pathogens/index.html) for PFGE of *E. coli* O157:H7, *Shigella*, and *Salmonella* with following modifications. Genomic DNAs (in agarose plugs) released from lysed cells of all isolates were digested with *Apa*I (40 U/μl) for 5 h at 30 °C. Samples were run on 1 % SeaKem Gold pulsed field agarose gels in 0.5 × TBE using a Bio-Rad CHEF-Mapper XA Electrophoresis System in 0.5 × TBE for 18 h with an initial pulse at 5 s and final pulse at 13 s. To compare the results of each isolate on different gels, *Salmonella braenderup* H9812 strain genomic DNA digested with *Xba* I was used as DNA size standard. The PFGE results were interpreted by categorizing gel lanes visually, according to guidelines described by Tenover and colleagues as used in our previous studies [14, 25, 27]. The genomic DNA banding patterns of the various isolates generated by PFGE were analyzed for clonal relationships using BioNumerics software, version 5.1 (Applied Maths, Austin, TX), using the UPGMA (unweighted pair group method with arithmetic mean) clustering method and the Dice similarity coefficient with 1.5 % band matching tolerance and 0.5 % optimization.

### Tri-locus multiplex PCR

Tri-locus multiplex PCR was performed using genomic DNA from these isolates to determine clonal relationships (Global Clones I, II or III). Briefly, six pairs of PCR primers designed by Turton and colleagues [28] were used to perform two sets of multiplex PCR amplifications of *ompA*, *csuE* and *bla*_OXA–51-like_ gene fragments for each of the isolates using 5 Prime Taq polymerase MasterMix (Gaithersburg, MD). PCR parameters consisted of an initial denaturation at 94 °C for 3 min, 30 cycles of denaturation at 94 °C for 45 s, annealing at 57 °C for 45 s, extension at 72 °C for 1 min, and a final extension at 72 °C for 5 min. The isolates were designated as *A. baumannii* Global Clones I, or II based on criteria established for Group 2 and Group 1 strains, respectively [28].

### Multi-locus sequence typing (MLST)

The MLST scheme described by Diancourt and coworkers [29] was performed and the scheme defined as Pasteur’s MLST scheme is now hosted at http://pubmlst.org/abaumannii/ site of PubMLST database. Briefly, the sequences of internal fragments of seven house-keeping genes *cpn60* (encoding 60-kDa chaperonin), *fusA* (protein elongation factor EF-G), *gltA* (the citrate synthase), *pyrG* (CTP synthase), *recA* (homologous recombination factor), *rplB* (50S ribosomal proteins L2) and *rpoB* (RNA polymerase subunit B) were determined. PCR amplification was performed in separate reactions of a final volume of 50 μl containing 20 μl Taq polymerase MasterMix, 1 μl forward and reverse primers (10 μM) and 2 μl template DNA (10 ng/μl). PCR amplification was carried out in the GeneAmp 9700 system without a mineral oil overlay. Thermocycler parameters included an initial melt at 94 °C for 2 min, followed by 35 cycles of 94 °C for 30 s, 50 °C for 30 s, 72 °C for 30 s, ending with a final extension step of 72 °C for 5 min. After confirmation of presence of robust amplicons at appropriate length, PCR products were purified according to the manufacturer’s instructions using a QIAquick PCR purification kit (Qiagen, Valencia, CA) and then sequenced on an ABI 3730 automated fluorescent sequencer. Determination of the sequence type was carried out using the Pasteur MLST Database on the http://pubmlst.org/abaumannii/ website.

### Antimicrobial susceptibility testing

Antimicrobial susceptibility of the *A. baumannii* isolates were determined using the broth microdilution protocols of Clinical Laboratory Standards Institute [30] against a total of 14 known antibiotics according to methods described previously [14]. Cefotaxime, ceftazidime, ceftriaxone, gentamicin, levofloxacin, ciprofloxacin, piperacillin and polymyxin B were purchased from Sigma-Aldrich (St. Louis, MO). Meropenem was from US Pharmacopeia (Rockville, MD). Amikacin, cefepime, gatifloxacin, imipenem and tobramycin were purchased from Fisher Scientific (Tustin, CA).

### Sequencing of QRDRs of *gyrA* and *parC* genes

The quinolone resistance determinant regions (QRDRs) of *A. baumannii* genes *gyrA* and *parC* were sequenced based on the methods described previously [14], with the exception of a new forward PCR/sequencing primer (5′-GTGTGCTTTATGCCATGCAC-3′) for the *gyrA* QRDR. Sequence comparisons with the amino acid sequences of wild type *A. baumannii* GyrA (accession No. X82165) and ParC (accession No. X95819) QRDR regions [31, 32] were made to identify mutated amino acids.

### Detection of class 1 integrons

The presence or absence of Class 1 integrons in all 38 hospital isolates was determined by PCR using previously reported primers specific for the *int*I1 genes: intI1L primer, 5′-ACATGTGATGGCGACGCACGA-3′; intI1R primer, 5′-ATTTCTGTCCTGGCTGGCGA-3′ [33]. Amplification was performed using genomic DNA as template as described previous for the QRDR of the *gyrA* and *parC* genes [14], with the following parameters: an initial template denaturation at 94 °C for 2 min; 36 cycles of 1 min denaturation at 95 °C, 30 s of annealing at 53 °C, and 2 min of extension at 72 °C; final extension at 72 °C for 10 min. Similarly, primers specific to the 5′ and 3′conserved segments (CS) of class 1 integrons (5′ CS primer, 5′-GGCATCCAAGCAGCAAG-3′; 3′ CS primer, 5′- AAGCAGACTTGACCTGA-3′) were also chosen to amplify the variable regions, as previously described by Levesque and coworkers [34]. After the PCR products were confirmed by agarose gel electrophoresis analysis, successful amplicons were sequenced as previously described for the QRDR of *gyrA* and *parC* genes. DNA sequences were analyzed using NCBI BLAST program to identify the entities of the genes within the variable regions.

### Carbapenamase gene detection

The presence of four prevalent OXA carbapenemase genes (*bl*a_OXA–23-like_, *bla*_OXA–24-like_, *bla*_OXA–51-like_ and *bla*_OXA–58-like_) for all isolates was determined using established procedures described by Woodford and colleagues [35]. Briefly, the multiplex PCR reactions using 4 pairs of published primers [35] were performed in the GeneAmp 9700 system. The resulting agarose gel electrophoresis revealed the presence or absence of a given *bla*_OXA_ allele based on the predicted amplicon sizes [35].

### Detection of IS*Aba1*

These hospital isolates were subsequently tested for the presence of IS*Aba1* element, using procedures described by Segal and coworkers [36]. Additionally, for those isolates that were confirmed to harbor *bla*_OXA–23_, PCR mapping experiments were performed to determine whether there was an IS*Aba1* element upstream of the *bla*_OXA–23_ gene using the IS*Aba1* forward primer and the *bla*_OXA–23-like_ gene reverse primer, 20 ng template DNA, with the following PCR parameters: an initial denaturing step at 94 °C for 5 min, followed by 30 amplification cycles of 94 °C for 25 s, 52 °C for 40 s and 72 °C for 50 s, with a final extension step at 72 °C for 6 min.

## Results and discussion

### Epidemiology

All isolates were confirmed as belonging to *A. baumannii* species based on sequence analysis of the 16S-23S rRNA intergenic spacer (ITS) region (data not shown). Outbreak X (*n* = 6 cases) and Outbreak Y (*n* = 8 cases) were defined as hospital-acquired *A. baumannii* infections clustered in time and location. Thirty-five *A. baumannii* clinical isolates, obtained from two large general acute care hospitals (>300 beds) as part of two respective outbreak investigations, were collected and analyzed from twenty-one unique patients. Specimen sites included respiratory (resp, sputum, endotracheal tube, and bronchoalveolar lavage) (*n* = 19), blood (*n* = 7), catheter tip (*n* = 4), wound (*n* = 4), and urine (*n* = 1) (Table 1). Three additional isolates were collected from the hospital environment in Outbreak Y (sink faucet, towel dispenser and ventilator tubing in the same room). Outbreak X occurred in the burn intensive care unit. All six cases (i.e., case patients) were admitted for severe thermal injuries. Outbreak Y occurred in the critical care unit of another hospital and involved cases who were admitted for a variety of medical diagnoses including respiratory failure, altered consciousness, dehydration, abdominal distress, end stage renal disease, and stroke. The remaining six patients in Outbreak X and the remaining patient in Outbreak Y were considered controls (i.e., control patients). The control patients in Outbreak X were admitted to intensive care units (*n* = 4) or closed monitored units (*n* = 2), a step-down unit from intensive care, for conditions that include wounds, cancer, trauma, sepsis, and necrosis. The control patient in Outbreak Y was admitted to the telemetry unit for fever and hypotension. For the twenty-one total patients, the average age was 56 years (ranging from 19 to 85 years); 76 % (*n* = 16) were male and 24 % (*n* = 5) were female; 48 % of patients died (*n* = 10) with cause of death determined to be related to *A. baumannii* in three cases. The average length of stay prior to date of first positive *A. baumannii* culture was 25 days (range 1–109 days).

### Pulsed field gel electrophoresis analysis

**Fig. 1:**
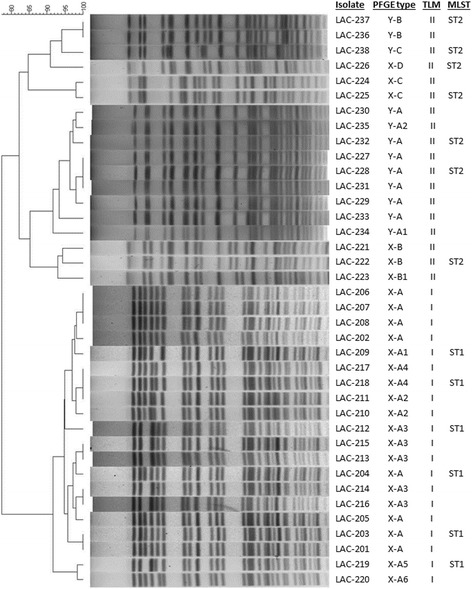
The dendrogram of PFGE profiles of the 38 *A. baumannii* isolates and comparison of PFGE types, trilocus multiplex PCR types and MLST sequence types. The dendrogram includes PFGE patterns of 38 isolates and the comparison was performed with the BioNumerics software v5.1 using the UPGMA (unweighted pair group method with arithmetic mean) clustering method and the Dice similarity coefficient with 1.5 % band matching tolerance and 0.5 % optimization. The PFGE types were assigned, first based on the outbreaks (X or Y), then based to the degree of similarity according to guidelines of Tenover and colleagues [27]

Pulsed field gel electrophoresis demonstrated relative relatedness of case isolates as compared to control isolates in each outbreak respectively. In Outbreak X, eight isolates from four cases (Cases 1–4) had indistinguishable isolate patterns and were designated Type X-A (“X” was added as a prefix to distinguish the PFGE types from those of Outbreak Y), while one isolate from Case 4 was designated Type X-A1 due to 1 band difference (Table 1 and Fig. 1 PFGE). The five isolates from the remaining two cases (Case 5 and Case 6) were designated Type X-A3, were indistinguishable from each other and had three band differences from the major pattern of the other cases (Table 1 and Fig. 1). Among the control isolates, two isolates of Control 1 and one isolate of Control 3 showed three band differences from the major pattern of the cases and were designated Type X-A2 and Type X-A5, respectively. Two isolates of Control 2 and the other isolate of Control 3 had four band differences from the major pattern and were designated Type X-A4 and Type X-A6, respectively (Table 1 and Fig. 1 PFGE). The isolates of the remaining three controls had greater than seven band differences from the major pattern of the cases, thus are considered not clonally related to those described above and were designated Types X-B, X-B1, X-C and X-D (Table 1 and Fig. 1). In Outbreak Y, four cases (Cases 1–4) were indistinguishable and designated Type Y-A (Table 1 and Fig. 1). One case (Case 5) and one control (Control 1) each had one band difference from the major pattern of the cases and were designated Types Y-A1 and Y-A2. All 3 patterns (Y-A, Y-A1, and Y-A2) are between 1–3 band apart so they are considered closely related but are not the same, according to Tenover et al [27]. Two cases (Case 6 and Case 7) were indistinguishable from each other but had greater than seven band differences from the other cases and were designated Type Y-B, and the remaining case (Case 8) was greater than seven band different from the major patterns of the cases and was designated Type Y-C (Table 1 and Fig. 1). The isolates with Types Y-B and Y-C are deemed not clonally related to Types Y-A to Y-A2. The three environmental isolates were also designated Type Y-A, and had 0 band difference from the major pattern of the cases (Table 1 and Fig. 1). Of note, in Outbreak X, one case (Case 4) and two controls (Control 3 and Control 5) had isolates collected from different sites on different days that, though they were from the same patient, differed from each other (Table 1). Among 38 isolates obtained from two hospital outbreaks, there were two clonally related clusters: one cluster consisting of isolates with Types X-A1 to X-A6 (*n* = 20) and the other cluster of isolates with Types Y-A to Y-A2 (*n* = 9) (Fig. 1). The remaining six isolates collected from Outbreak X were all from controls, while the remaining three isolates from Outbreak Y were from three cases.

### Typing of the isolates based on trilocus multiplex PCR and MLST

To determine which Global Clone (previously known as European Clone or International Clone) each of the *A. baumannii* isolates belongs to, trilocus multiplex (TLM) PCR was performed. Our results showed that, consistent with PFGE profiles (Fig. 1), 20 isolates associated with Outbreak X (14 case isolates, 6 control isolates) were found to belong to Global Clone I and they are all clonally related based on PFGE patterns (Fig. 1). In contrast, all 12 isolates from Outbreak Y (including case, control and environmental isolates) were found to belong to Global Clone II and all but three (LAC-236. LAC-237 and LAC-238) were clonally related (Fig. 1). Interestingly, six control isolates from three control patients in Outbreak X were also typed to Global Clone II (Fig. 1).

To confirm TLM typing results, 13 representative *A. baumannii* isolates from the outbreaks were examined for their MLST sequence types (ST) based on the Pasteur Institute Scheme [29]. The MLST results with select isolates showed those isolates typed to Global Clone I were confirmed to be ST1 while those isolates typed to Global Clone II were confirmed to be ST2 (Fig. 1). These results confirmed that for these isolates, TLM PCR assays accurately typed them to respective Global Clones, consistent with their sequence typing. In summary, the first outbreak (X) involved the spread of 20 isolates assigned to PFGE types X-A to X-A6 which belong to ST-1, the second outbreak (Y) resulted in the spread of 9 isolates assigned to PFGE types Y-A to Y-A2 which belong to ST-2. Isolates with Y-B, Y-C patterns are not related to Y-A, Y-A1 and Y-A2 isolates even though they were also typed ST-2.

MLST, TLM and PFGE genotyping methods are frequently used for epidemiological studies and the surveillance of *A. baumannii* outbreaks [37, 38]. PFGE has been considered the gold standard for outbreak investigations due to its higher discriminatory power. For years, PFGE had been the leading standard for typing of *A. baumannii* isolates [38]. However issues related to interlab exchange of data and reproducibility has raised concerns about reliability and portability of the results. While the TLM approach is simple to perform and has higher throughput, it has failed to resolve strains belonging to unusual lineages [24]. On the other hand, MLST is highly discriminating and uses the DNA sequences of several conserved housekeeping gene fragments [39]. It has emerged as the preferred method to type *A. baumannii* [40–42]. Our experience indicates that a bipartite interrogation using both PFGE and MLST approaches is ideal in examination of molecular epidemiology of *A. baumannii* isolates.

### Antimicrobial susceptibility

To determine the antimicrobial susceptibility of these *A. baumannii* isolates, minimal inhibitory concentrations (MICs) were determined for a panel of 14 antibiotics against the 38 isolates obtained from the two outbreaks in Los Angeles County hospitals. Based on the MICs obtained for each antibiotic for the two groups of isolates (Outbreaks X and Y), MIC range, MIC_50_ and MIC_90_ values are summarized in Table 2. With the exception of polymyxin B which is active against all isolates, MIC_50_ values of all antibiotics tested fell within resistant ranges as defined by CLSI [43]. Specifically, all 38 isolates were resistant to cefepime, cefotaxime, ceftazidime, ceftriaxone, ciprofloxacin, levofloxacin and piperacillin (based on MIC ranges, Table 2), some may even be called XDR (extremely drug-resistant) (data not shown) as defined by Magiorakos and colleagues [44]. Additionally, while the X group of isolates (outbreak X) appeared to be more heterogeneous than those of Y group (outbreak Y) because of higher number of antibiotics with wide range of MIC values, the MIC_50_ and MIC_90_ profiles were quite similar between the two groups (Table 2).

**Table 2:** MIC ranges, MIC_50_ and MIC_90_ values based on outbreak

Outbreak	Antimicrobial agents	MIC, μg/ml		
		range	MIC_50_	MIC_90_
Outbreak X (*n* = 26)	Amikacin	1–256	256	256
	Gentamicin	4–256	256	256
	Tobramycin	0.5–256	256	256
	Imipenem	2–32	16	32
	Meropenem	8–64	32	64
	Piperacillin	256	256	256
	Cefepime	64–256	128	256
	Cefotaxime	256	256	256
	Ceftazidime	256	256	256
	Ceftriaxone	256	256	256
	Ciprofloxacin	64–256	256	256
	Gatifloxacin	4–64	32	64
	Levofloxacin	16–32	32	32
	Polymyxin B	0.25–0.5	0.25	0.5
Outbreak Y (*n* = 12)	Amikacin	256	256	256
	Gentamicin	256	256	256
	Tobramycin	256	256	256
	Imipenem	1–32	32	32
	Meropenem	2–32	32	32
	Piperacillin	256	256	256
	Cefepime	32	32	32
	Cefotaxime	256	256	256
	Ceftazidime	128–256	256	256
	Ceftriaxone	256	256	256
	Ciprofloxacin	64–128	128	128
	Gatifloxacin	8–64	16	32
	Levofloxacin	16–64	32	64
	Polymyxin B	0.25	0.25	0.25

There have been few reports on molecular epidemiology and antimicrobial resistance of isolates of *A. baumannii* obtained from hospitals in Los Angeles metropolitan area. In one of such reports, we characterized genetic and antimicrobial resistance profiles of 20 representative outbreak isolates of *A. baumannii* derived from hospitals in Los Angeles County, CA [14]. Using PFGE technique, these 20 multidrug resistant *A. baumannii* isolates were classified into eight epidemiologically distinct groups, which were consistent with isolates’ antimicrobial susceptibility profiles [14]. Armed with more sophisticated molecular typing methodologies, such as MLST, we recently typed nine of these LAC isolates to ST10 (including LAC-4), five of them to ST417, two of them to ST241, while four of them confirmed as ST2 [24]. Although all isolates from the present study belong to ST1 and ST2 thus may not be genetically related to those 20 isolates examined previously, the increase in resistance phenotypes of the 38 isolates is quite remarkable (Table 2), considering the two batches of isolates were collected ten years apart. For example, all of the case isolates in the present study were resistant to all three aminoglycoside antibiotics tested (data not shown), while 25 % of isolates (5/20) from the previous batch were susceptible to amikacin and tobramycin [14]. Secondly, with the exception of three isolates (LAC-236, –237, and –238), all case isolates from the present study were resistant to both carbapenems (data not shown); in contrast, 50 % and 45 % of isolates from previous batch were susceptible to imipenem and meropenem, respectively [14].

Recently, Miyasaki and coworkers examined in vitro activity of antibiotic combinations against a panel of multidrug resistant strains of *A. baumannii* collected from Cedars-Sinai Medical Center in Los Angeles, CA [45]. In their report, among 102 hospital-acquired MDR *A. baumannii* isolates, 76 belonged to a major clone A while eight to a minor clone B, as characterized by repetitive PCR amplification using the DiversiLab *Acinetobacter* Fingerprinting Kit [45]. Unfortunately, because MLST tests were not performed by Miyasaki and colleagues, the genetic relationships of their isolates to those described in our studies (Fig. 1) [14] are unknown. In a study that reports immuno-protection against XDR *A. baumannii* infection in mice, Luo and coworkers described five isolates collected from in-patients at Harbor-UCLA Medical Center, Los Angeles, CA, in 2009 [46]. These five isolates were found to exhibit resistance phenotypes to at least one antimicrobial agents of all but 2 categories of antimicrobial agents [46].

### Mechanisms of resistance

Our antimicrobial susceptibility testing indicated that all isolates were resistant to the three fluoroquinolone (FQ) antibiotics, with the exception of one control isolate which was intermediate to gatifloxacin (data not shown). Point mutations in the QRDR of genes encoding the GyrA and ParC subunits of DNA gyrase and DNA topoisomerase IV, respectively, are the primary mechanism of high-level bacterial resistance to fluoroquinolones (FQs) [31, 32]. These mutations and the resultant amino acid changes led to an altered target protein structure and reduced fluoroquinolones binding affinity [47]. To confirm if this is the case for these *A. baumannii* isolates, the QRDRs of the *gyrA* and *parC* genes of the 38 *A. baumannii* isolates were amplified via PCR and sequenced. Sequencing results indicated that with exceptions of control isolates LAC-222 and LAC-225, all the remaining 36 isolates possess point mutations that led to amino acid substitutions in both GyrA and ParC polypeptides (Table 3). In LAC-222, only one mutation that occurred in *gyrA* gene was found, which explains the intermediate phenotype of LAC-222 to gatifloxacin (data not shown).

**Table 3:** GyrA and ParC amino acid substitutions and detection of Class 1 integrons in *A. baumannii* isolates

Isolate No.	GyrA amino acid changes			ParC amino acid changes			Class 1 integron		*bla* gene or IS*Aba1* PCR					
	Gly -81	Ser-83 TCA	Ala-84	Glu-87	Ser-80 TCG	Glu-84	integrase	cassette organization	OXA-23	OXA-24	OXA-51	OXA-58	IS*Aba1*	IS/OXA-23 PCR
LAC–201	–	Leu TTA	–	–	Leu TTG	–	+	*aacA4–catB8–aadA1*	+	–	+	–	+	+
LAC–202	–	Leu TTA	–	–	Leu TTG	–	+	*aacA4–catB8–aadA1*	+	–	+	–	+	+
LAC–203	–	Leu TTA	–	–	Leu TTG	–	+	*aacA4–catB8–aadA1*	+	–	+	–	+	+
LAC–204	–	Leu TTA	–	–	Leu TTG	–	+	*aacA4–catB8–aadA1*	+	–	+	–	+	+
LAC–205	–	Leu TTA	–	–	Leu TTG	–	+	*aacA4–catB8–aadA1*	+	–	+	–	+	+
LAC–206	–	Leu TTA	–	–	Leu TTG	–	+	*aacA4–catB8–aadA1*	+	–	+	–	+	+
LAC–207	–	Leu TTA	–	–	Leu TTG	–	+	*aacA4–catB8–aadA1*	+	–	+	–	+	+
LAC–208	–	Leu TTA	–	–	Leu TTG	–	+	*aacA4–catB8–aadA1*	+	–	+	–	+	+
LAC–209	–	Leu TTA	–	–	Leu TTG	–	+	*aacA4–catB8–aadA1*	+	–	+	–	+	+
LAC–210	–	Leu TTA	–	–	Leu TTG	–	–		+	–	+	–	+	+
LAC–211	–	Leu TTA	–	–	Leu TTG	–	–		+	–	+	–	+	+
LAC–212	–	Leu TTA	–	–	Leu TTG	–	+	*aacA4–catB8–aadA1*	+	–	+	–	+	+
LAC–213	–	Leu TTA	–	–	Leu TTG	–	+	*aacA4–catB8–aadA1*	+	–	+	–	+	+
LAC–214	–	Leu TTA	–	–	Leu TTG	–	+	*aacA4–catB8–aadA1*	+	–	+	–	+	+
LAC–215	–	Leu TTA	–	–	Leu TTG	–	+	*aacA4–catB8–aadA1*	+	–	+	–	+	+
LAC–216	–	Leu TTA	–	–	Leu TTG	–	+	*aacA4–catB8–aadA1*	+	–	+	–	+	+
LAC–217	–	Leu TTA	–	–	Leu TTG	–	–		+	–	+	–	+	+
LAC–218	–	Leu TTA	–	–	Leu TTG	–	+	*aacA4–catB8–aadA1*	+	–	+	–	+	+
LAC–219	–	Leu TTA	–	–	Leu TTG	–	–		+	–	+	–	+	+
LAC–220	–	Leu TTA	–	–	Leu TTG	–	–		+	–	+	–	+	+
LAC–221	–	Leu TTA	–	–	Leu TTG	–	+	*aacA4–catB8–aadA1*	–	–	+	–	+	
LAC–222	–	Leu TTA	–	–	–	–	+	*aacA4–catB8–aadA1*	–	–	+	–	+	
LAC–223	–	Leu TTA	–	–	Leu TTG	–	+	*aacA4–catB8–aadA1*	–	–	+	–	+	
LAC–224	–	Leu TTA	–	–	Leu TTG	–	–		–	–	+	–	+	
LAC–225	–	Leu TTA	–	–	–	–	–		–	–	+	–	+	
LAC–226	–	Leu TTA	–	–	Leu TTG	–	+	*aacA4–catB8–aadA1*	–	–	+	–	+	
LAC–227	–	Leu TTA	–	–	Leu TTG	–	+	*aacA4–catB8–aadA1*	+	–	+	–	+	+
LAC–228	–	Leu TTA	–	–	Leu TTG	–	+	*aacA4–catB8–aadA1*	+	–	+	–	+	+
LAC–229	–	Leu TTA	–	–	Leu TTG	–	+	*aacA4–catB8–aadA1*	+	–	+	–	+	+
LAC–230	–	Leu TTA	–	–	Leu TTG	–	+	*aacA4–catB8–aadA1*	+	–	+	–	+	+
LAC–231	–	Leu TTA	–	–	Leu TTG	–	+	*aacA4–catB8–aadA1*	+	–	+	–	+	+
LAC–232	–	Leu TTA	–	–	Leu TTG	–	+	*aacA4–catB8–aadA1*	+	–	+	–	+	+
LAC–233	–	Leu TTA	–	–	Leu TTG	–	+	*aacA4–catB8–aadA1*	+	–	+	–	+	+
LAC–234	–	Leu TTA	–	–	Leu TTG	–	+	*aacA4–catB8–aadA1*	+	–	+	–	+	+
LAC–235	–	Leu TTA	–	–	Leu TTG	–	+	*aacA4–catB8–aadA1*	+	–	+	–	+	+
LAC–236	–	Leu TTA	–	–	Leu TTG	–	+	*aacA4–catB8–aadA1*	–	–	+	–	+	
LAC–237	–	Leu TTA	–	–	Leu TTG	–	+	*aacA4–catB8–aadA1*	–	–	+	–	+	
LAC–238	–	Leu TTA	–	–	Leu TTG	–	+	*aacA4–catB8–aadA1*	–	–	+	–	+	

Absence of mutations in *gyrA* or *parC* genes is denoted as “–“

Presence or absence of class 1 integrase gene is denoted as “+” or “–”

Empty spaces, no tests were performed

These results are consistent with the previous findings that mutations in both GyrA and ParC are required for high-level FQ resistance phenotypes [31]. Indeed, we have previously reported that presence of mutations in both *gyrA* and *parC* genes in *A. baumannii* isolates is associated with high-level resistance to fluoroquinolones [14, 25]. Similarly, other investigators have found that possessing mutations in both *gyrA* and *parC* genes is strongly correlated with high level resistance to ciprofloxacin in *A. baumannii* isolates [18] or *A. pittii* isolates [48].

To assess the role of Class 1 integrons and the associated antibiotic resistance gene cassettes in antibiotic resistance phenotypes in the isolates, we performed PCR screens against the 38 isolates for the presence of Class 1 integrons by amplifying the integrase gene. Our results revealed that 31 of the 38 isolates harbored the integrase gene (Table 3) and that the seven isolates without integrase gene were all derived from control patients (Table 1 and Table 3). Subsequent conserved segment amplification via PCR, followed by DNA sequencing, indicated that all amplified conserved segments contain three antibiotic resistance genes sharing the same gene identity and organization: *aacA4, catB8* and *aadA1* (Table 3). Since *aacA4* [aka *aac (6’)-Ib*] encodes aminoglycoside 6’-*N*-acetyltransferase, which confers resistance to amikacin and tobramycin, among others [49, 50], the presence of this gene (Table 3) correlates well with the susceptibility patterns of these 38 *A. baumannii* isolates because four of the seven control isolates being negative for class 1 integron were found to be susceptible to at least two of the three aminoglycoside antibiotics (data not shown). The presence of the integrase gene in 31 of the 38 isolates provides a critical surveillance parameter that is indicative of acquisition of antibiotic resistance genes.

Integrons have been shown to be an important type of mobile elements contributing significantly to the dissemination of antibiotic resistance genes in Gram-negative bacterial pathogens [51, 52]. It was interesting to note that the majority of *A. baumannii* clinical isolates tested in this study harbor Class 1 integron in their genomes and the seven isolates without it were control isolates, indicating that the control isolates had not yet acquired the Class 1 integron. The fact that outbreak isolates from two hospitals shared that same antibiotic resistance gene array suggests the prevalence of this particular type of Class 1 integron in Los Angeles area. Such a gene array, *aacA4-catB8-aadA1*, was also found in many MDR *A. baumannii* clinical isolates collected from prison inmates [25]. This same array of gene cassettes has been identified in clinical isolates of *A. baumannii* worldwide: in 25 of the 30 *A. baumannii* isolates in Korea [53], in 47 of the 65 *A. baumannii* isolates in Taiwan [54], in all 30 clonal isolates of carbapenem-resistant *A. baumannii* involved in a hospital outbreak in Latvia [55], and in 26 clinical isolates of *A. baumannii* obtained in central Ohio, U.S.A. [56].

Carbapenem resistance in *A. baumannii* has been suggested to contribute to increased risk of mortality in patients infected with this bacterium [57]. In this study, the majority of *A. baumannii* isolates tested were resistant to both imipenem and meropenem, with a few isolates exhibiting intermediate or susceptible phenotypes, suggesting widespread resistant strain dissemination in the LAC hospital setting. Multiplex PCR reactions using primer pairs specific to four OXA carbapenemase genes (*bl*a_OXA–23-like_, *bla*_OXA–24-like_, *bla*_OXA–51-like_ and *bla*_OXA–58-like_) were performed against these 38 isolates. As anticipated, *bla*_OXA–51-like_ gene was detected in every *A. baumannii* isolates (Table 3). Additionally, 29 out of 38 isolates (76 %) also harbor the *bl*a_OXA–23-like_ gene (Table 3). Furthermore, none of the isolates harbors *bla*_OXA–24-like_ or *bla*_OXA–58-like_ gene. Results from the IS*Aba1* element PCR screens indicated that IS*Aba1* was present in all of the 38 isolates (Table 3). Subsequent PCR screens against the 29 *bl*a_OXA–23_ positive strains using IS*Aba1* forward and *bl*a_OXA–23_ reverse primers confirmed that all of these 29 isolates possess IS*Aba1* element upstream of the *bl*a_OXA–23_ gene (Table 3), which is consistent with the carbapenem resistant phenotypes of these isolates. The remaining nine isolates without the *bl*a_OXA–23_ gene were either susceptible or intermediate to at least one carbapenem tested (data not shown). Remarkably, 20 isolates of the clonal cluster (LAC-201 to –220) from Outbreak X and nine isolates of the clonal cluster (LAC-227 to –235) from Outbreak Y all possess IS*Aba1*-linked *bl*a_OXA –23_ gene (Fig. 1, Table 3) and they all are resistant to both carbapenems (data not shown).

Carbapenems are considered the last resort to treat infections caused by *A. baumannii*. In this study, we showed that the majority of case isolates and some control isolates were resistant to both carbapenems, which is of great concern. The carbapenem susceptibility phenotypes in these 38 *A. baumannii* isolates are clearly correlated with the presence or absence, in the genomes of the isolates, of IS*Aba1*-linked *bla*_OXA –23-LIKE_ genes. Presumably, the IS*Aba1* element likely provides exogenous promoter functions to over-express the linked genes, as demonstrated previously [41, 58, 59]. In contrast, among the 20 nosocomial outbreak isolates of *A. baumannii* reported previously by us, 50 % and 45 % were susceptible to imipenem and meropenem, respectively [14], suggesting rapid dissemination of carbapenem resistant genetic determinants over a ten-year span. In fact, the LAC-4 strain, whose genome was completely sequenced, and 19 other LAC isolates from the 2008 study all lack *bl*a_OXA –23-like_, *bla*_OXA –24-like_, and *bla*_OXA –58-like_ genes, even though IS*Aba1* was detected in all of these isolates (data not shown). On the other hand, MDR *A. baumannii* strains isolated recently were often found to be resistant to carbapenems. For example, Hammerum and coworkers described eight carbapenem-resistant *A. baumannii* isolates in a Denmark hospital. These strains were typed using Pasteur MLST Scheme to five ST2 isolates, two ST1 isolates and one ST158, all of which harboring either *bl*a_OXA –23_ or *bla*_OXA –72_ (which is *bla*_OXA –24-LIKE_) genes, consistent with carbapenem resistance phenotype [40]. Additionally, 26 carbapenem-resistant *A. baumannii* clinical isolates from two Italian hospitals were characterized and typed to ST2 using MLST and all possess *bl*a_OXA –82_ genes that are located downstream of IS*Aba1* elements [60]. Finally, Teo and coworkers identified 49 MDR *A. baumanniii* clinical isolates that were resistant to carbapenems and all isolates were found to harbor both *bl*a_OXA –51_ and *bla*_OXA –23_ genes, in accordance with carbapenem resistant phenotypes [61].

## Conclusions

This report presents the phenotypic and molecular analyses of 38 isolates of *A. baumannii* collected from two hospital outbreaks in Los Angeles County. Based on genetic patterns and molecular tests, one outbreak (Outbreak X) was dominated by a cluster of 20 clonally related isolates derived from Global Clone I or ST1 (MLST Pasteuar Scheme) while the other (Outbreak Y) was dominated by a different cluster of nine clonally related strains belonging to Global Clone II or ST2. While every single isolate (including environmental isolates from the hospital) is multidrug resistant, all isolates were found uniformly resistant to seven antibiotics belonging to cephalosporin, penicillin and fluoroquinolone classes. There was good correlation between antimicrobial susceptibility phenotypes and the presence of resistance genetic determinants. In particular, it is alarming that nearly all of these cluster isolates (except three that were obtained from control patients) were resistant to both imipenem and meropenem, which can be attributed to presence of *bl*a_OXA –23_ genes downstream of the IS*Aba1* in the genomes of these isolates. The results presented here provide clinically relevant insights into antimicrobial susceptibility, clonal dissemination and mechanisms of resistance in Los Angeles area hospitals.

### Ethics approval and consent to participate

Because the clinical isolates were not isolated by coauthors who had no direct interactions with patients from whom these isolates were isolated and the isolates do not contain patient identifiable information, ethics review and approval by institutional review board (IRB) was waived. Consent to participate is not applicable.

### Consent for publication

Not applicable.

### Availability of data and materials

All data is contained within the manuscript. Clinical isolates of *A. baumannii* will be made available upon requests from Dr. H. Howard Xu.

## Abbreviations

ATCC: American type culture collection
BAL: bronchoalveolar lavage
BLAST: basic local alignment search tool
ETT: endotracheal tube
FQ: fluoroquinolone
ITS: intergenic spacer
LAC: Los Angeles County
MDR: multidrug resistance
MIC: minimal inhibitory concentration
MLST: multilocus sequence typing
NCBI: national center for biotechnology information
PCR: polymerase chain reaction
PFGE: pulsed field gel electrophoresis
QRDR: quinolone resistance determinant region
ST: sequencing type
TBE: tris borate EDTA buffer
TLM-PCR: trilocus multiplex PCR
UPGMA: unweighted pair group method with arithmetic mean
XDR: extreme drug resistance
